# Comparison of Baroreflex Sensitivity and Cardiac Autonomic Function Between Adolescent Athlete and Non-athlete Boys – A Cross-Sectional Study

**DOI:** 10.3389/fphys.2019.01043

**Published:** 2019-08-22

**Authors:** Senthil Kumar Subramanian, Vivek Kumar Sharma, Vinayathan Arunachalam, Rajathi Rajendran, Archana Gaur

**Affiliations:** ^1^Department of Physiology, All India Institute of Medical Sciences (AIIMS), Mangalagiri, Vijayawada, India; ^2^Department of Physiology, Government Institute of Medical Sciences, Greater Noida, India; ^3^Jawahar Navodaya Vidyalaya, Pondicherry, India; ^4^Department of Physiology, Jawaharlal Institute of Postgraduate Medical Education and Research, Pondicherry, India; ^5^Department of Physiology, Chengalpattu Medical College, Chengalpattu, India

**Keywords:** heart rate variability, school going, children, baroreflex sensitivity, 30:15 ratio, EI ratio, physical activity

## Abstract

**Introduction:** It is well known that regular physical activity improves cardiovascular health, and higher baroreflex sensitivity and heart rate variability are associated with cardiovascular health. Adolescence is the age when an individual’s behavior is easily modified; early intervention at this stage in terms of physical conditioning or training prevents future cardiovascular risk. Hence, we conceived the present study to assess and compare the baroreflex sensitivity and autonomic function between adolescent athletes and non-athletes.

**Methods:** We recruited school going athletes (*n* = 30) and non-athlete boys (*n* = 30) in the 10–19 age group after obtaining their assent and consent from their parents. We assessed height, weight, heart rate, blood pressure, baroreflex sensitivity, and cardiac autonomic function. Comparison between groups was made using the unpaired *t*-test for height, weight, body mass index, heart rate, blood pressure, and baroreflex sensitivity and using Mann-Whitney U test for cardiac autonomic function parameters.

**Results:** There was a trend for higher baroreflex sensitivity in athletes. Heart rate variability (total power and SDNN) was higher in athletes. The parasympathetic tone was higher in terms of higher RMSSD, and higher HF power. Parasympathetic reactivity was higher in athletes in terms of higher 30:15 ratio and EI ratio.

**Conclusion:** Athletic level physical conditioning has a positive influence on baroreflex function and autonomic function that may prove beneficial to the adolescents’ cardiovascular health.

## Introduction

Sedentary lifestyle increases the incidence and prevalence of chronic diseases, especially cardiovascular diseases. WHO predicted that, by 2020, cardiovascular disease will be the leading cause of death. The prevalence of cardiovascular diseases in adolescents is higher in developing countries. Physical activity not only improves physical fitness but also reduces cardiometabolic diseases (Hu et al., 2001). Herein, it is crucial to assess cardiovascular risk among sedentary adolescents, and the effect of physical activity in this age group, which can address the root cause for escalating cardiovascular risk among adolescents.

Baroreceptor reflex mechanism plays a vital role in the short-term regulation of arterial pressure, by the chronotropic effect on the heart and by reflex regulation of parasympathetic and sympathetic outflow to bring about homeostasis. Reduced baroreflex sensitivity (BRS) is associated with hypertension (Mussalo et al., 2002), obesity, diabetes, and metabolic syndrome (Skrapari et al., 2006) in adults. Similarly, in adolescents, reduced BRS is associated with obesity (Lazarova et al., 2009), essential hypertension (Krontoradova et al., 2008), white-coat hypertension (Honzikova and Fiser, 2009), and insulin resistance (Honzikova and Zavodna, 2016). Honzíková et al. (2006) observed reduced BRS even in children and suggested that reduced BRS could be an etiology for hypertension rather than the result of it. Lantelme et al. (2002) showed that BRS correlates well with cardiometabolic risk factors such as age, SBP, DBP, pulse pressure, serum cholesterol, LDL cholesterol, serum triglycerides, and blood glucose. BRS integrates the consequences of these risk factors at various levels and hence might be a comprehensive cardiovascular risk factor (Lantelme et al., 2002) that could be used to predict future cardiovascular events.

Oscillations in the heart rate can be evaluated by heart rate variability (HRV), which is a non-invasive tool to assess the sympathetic and vagal influence on the heart. Available evidence suggests that assessment of BRS and heart rate variability reflects the cardiovascular risk of an individual (La Rovere et al., 2013). Decreased heart rate variability in adolescents is associated with obesity (Guizar et al., 2005), hypertension (Gui-Ling et al., 2013), psychiatric disorders (Blom et al., 2010), diabetes (Wawryk et al., 1997), and developmental changes (Seifert et al., 2014). Documenting the BRS and cardiac autonomic function in adolescents would help us in quantifying their future cardiovascular risk. Further, comparing BRS and cardiac autonomic function between athletes and non-athletes would help us to study the effect of physical activity on these parameters. Lifestyle modifications such as weight reduction (Alvarez et al., 2005) and physical activity (Loimaala et al., 2003) are reported to improve BRS values and cardiac autonomic function (Poirier et al., 2003; Nagai and Moritani, 2004). Adolescence is the age when an individual’s behavior can be easily modified, and early intervention at this stage in terms of physical conditioning or training is vital to prevent future cardiovascular risk. Hence, the present study was conceived with the intent to assess and compare the BRS and cardiac autonomic battery of tests between adolescent athletes and non-athletes.

## Materials and Methods

### Study Design

This was a cross-sectional study conducted in Department of Physiology, JIPMER, Puducherry, India, in collaboration with CBSE board schools in Puducherry. The study commenced after obtaining approval from the Institute Ethics Committee for Human Studies (No. JIP/IEC/2013/3/177).

### Participants

We considered boys aged 10–19 years, studying in CBSE schools in Pondicherry for the study. Students with a history of cardiovascular, respiratory, or organic disorder which prevents subjects from doing maximal exercise, or on any drugs that could affect vascular and/or autonomic functions or suffering from any acute illness were excluded from the study. We obtained informed written consent from the guardians/parents and written assent from the boys who had met the inclusion criteria. We recruited 30 boys representing their school at state, national, or international level aerobic sports and have undergone athletic level physical conditioning for at least 1 year (Group 1), and 30 age-matched non-athlete students (students participating in recreational sports activities but not in any inter-school athletic events for at least 1 year) were recruited as controls (Group 2).

### Parameters Measured

All the participants were requested to report to Autonomic function testing lab, Department of Physiology, JIPMER, Pondicherry, between 8 am and 11 am. We requested the participants to abstain from doing strenuous exercise, and taking caffeinated/alcoholic beverages 12 h before the recording and advised them to have good sleep the previous day of recording. The lab temperature was maintained at ±24°C and dimly lit. Participants were oriented toward the lab, and recording parameters were explained. Demonstration and practice sessions were given for all the procedures before starting the actual recording to alleviate their anxiety. Participants with poor sleep quality, flu, or minor ailments were requested to report on a later date to the recording. On the day of recording, participants were requested to report with an empty bladder, loose clothing, and we instructed them to have their breakfast 2 h before testing.

#### Anthropometric Parameters

Anthropometric parameters were measured by the International Society for the Advancement of Kinanthropometry (ISAK)-certified investigator. A wall-mounted stadiometer (VM Electronics Hardware Ltd) was used to measure the height accurate to the nearest 0.1 cm. Weight was measured using a digital weighing scale (Charder Electronic Co. Ltd. Taichung, Taiwan, 2013) accurate to the nearest 0.1 kg. Body mass index (BMI) was calculated using the Quetelet index – weight (kg)/height^2^ (m^2^).

#### Baseline Cardiovascular Parameters

Blood pressure (BP) and heart rate (HR) were measured after 10 min of rest in the sitting position. Heart rate was measured manually from the right radial artery. The BP (mm Hg) was recorded from the right arm using mercury sphygmomanometer (Diamond, Industrial-Electronic & allied products, Maharashtra, India). Recordings were taken thrice with 2-min rest intervals, and the average was taken as the final reading. All measurements were taken by the same investigator.

#### Baroreflex Sensitivity

We measured spontaneous BRS/resting BRS (spontaneous BP changes and HR changes that occur due to respiration) noninvasively by recording pulse rate and finger pressure using Finometer PRO (Finapres Medical Systems BV, Amsterdam, The Netherlands). Following 10 min of supine rest, the finger arterial pressure cuff and brachial artery cuff were placed in the middle finger and 2 cm above the elbow, respectively. Finger arterial pressure waveform was continuously monitored using volume clamp method of Penaz and the Physiocal criteria of Wesseling. Height correction is done using Height correction unit to remove hydrostatic errors caused by the small changes in finger position. Brachial artery pressure (BAP) waveform and level were reconstructed (reBAP) from finger pressure waveform using generalized waveform inverse modeling (Gizdulich et al., 1996, 1997).

Further, individual Riva-Rocci arm cuff return-to-flow pressure level calibration was done and added to reBAP to reduce the inaccuracies in representing the brachial artery pressure (Bos et al., 1996). Pulse rate and interbeat interval (IBI) were calculated from the finger pressure waveform. Personal computer-based data acquisition system Beatscope® Easy (Finapres Medical Systems BV, Amsterdam, The Netherlands) was used to view the data and to calculate BRS. BRS was obtained using time domain cross-correlation method. In this method, heartbeats are spline interpolated on a time scale having 1 s. The cross-correlation function is computed over a sliding correlation window of 12 beats with minimal overlap of five beats at various delays (0–5 s) between blood pressure and pulse interval. Then, the delay with the highest correlation is selected and when its coefficient of determination (*r*^2^) is significant (*p* < 0.05), it is accepted as BRS estimate (Westerhof et al., 2004).

#### Short-Term Heart Rate Variability

Short-term heart rate variability (HRV) was recorded following Taskforce on Heart Rate Variability-Standards of Measurement, Physiological Interpretation, and Clinical Use. (Task Force of the European Society of Cardiology the North American Society of Pacing Electrophysiology, 1996). Subject’s skin over the trunk was prepared using abrasive skin preparation gel Nuprep (ELprep, BIOPAC Systems, Inc., CA 93117, USA) for electrode placement. Then disposable EL503 snap electrodes for electrocardiography (ECG) (BIOPAC Systems, Inc., CA 93117, USA) were placed. Electrodes were connected to single-channel ECG100C – electrocardiogram amplifier module (BIOPAC Systems, Inc., CA 93117, USA) which in turn is connected to BIOPAC MP150A-CE Data acquisition unit through Universal interface module UIM100C (BIOPAC Systems, Inc., CA 93117, USA).

After 5 min of supine rest, lead II electrocardiography (ECG) was recorded for 5 min at a sampling rate of 1,000 Hz. The data are visualized using AcqKnowledge® software version 4.1 (BIOPAC Systems, Inc., CA 93117, USA) in Windows-based PC. Ectopics and artifacts were removed manually from the recorded ECG, and RR intervals of 5-min epoch from the lead II ECG recorded are taken in text format. From the RR tachogram, HRV analysis was done using Kubios version 1.0 (Bio-signal analysis Group, Finland). The frequency domain analysis and time domain analysis were computed using Fast Fourier Transformation (FFT) and RR trend, respectively. The frequency domain indices computed were very low frequency (VLF; 0.003–0.04 Hz), low frequency (LF; 0.04–0.15 Hz), and high frequency (HF; 0.15–0.4 Hz), both in absolute powers given as ms^2^ and in normalized unit (nu) [LFnu = LF/(TP − VLF) × 100 and HFnu = HF/(TP − VLF) × 100 or HFnu = 1 − LFnu], Total power (TP) ms^2^ (TP = VLF + LF + HF) and the LF/HF ratio. The time domain measures include standard deviation of all NN intervals (SDNN), the sum of the squares of differences between adjacent RR intervals (RMSSD), adjacent RR interval differing by more than 50 ms (NN50) and its percentage (pNN50).

Respiration was recorded as a separate channel through the same polygraph with appropriate recommended settings. Respiratory transducer belt (TSD201, BIOPAC Systems, Inc., CA 93117, USA) was placed over the abdomen and connected to respiratory amplifier module (RSP100C, BIOPAC Systems, Inc., CA 93117, USA) and connected to universal interface module UIM100C. Abnormalities concerning the frequency of breathing were carefully analyzed during and after the recording. Any respiratory tracing showing a frequency of more than 24 breaths per min, i.e., 0.4 Hz, was destined to be removed. Exceptional data, if any, were specifically examined and a decision is made regarding the inclusion of the HRV of that subject. However, in this study, such exceptional data were not encountered.

#### Autonomic Reactivity Tests

Lead II ECG was continuously monitored and recorded when necessary during the following procedures.

##### Forced Timed Breathing

We asked the subjects to perform forced timed breathing (FTB) at six breaths per minute, comprising inspiratory and expiratory cycles for 5 s each in the supine position (Novak, 2011). We instructed the subjects to avoid sharp inhalation/exhalation/holding of breath during the procedure. Deep breathing was synchronized to a paced voice metronome and, if necessary, guided by hand movement by the investigator. The ratio between maximal RR interval during expiration (E) and minimal RR interval during inspiration (I) is taken as E:I ratio.

##### Orthostatic Stress Test

Participants were asked to stand within 3 s from their supine position (Low, 2004). The ratio of the longest RR interval around the 30th beat and shortest around 15th beat (30:15 ratio) was calculated to obtain heart rate response to orthostatic stress test (OST).

##### Isometric Handgrip Test

Participants were made to sit comfortably in the couch. Maximal voluntary contraction during sustained isometric handgrip by the boys was measured using handgrip dynamometer (Inco, Ambala, India). Isometric handgrip (IHG) test was evaluated at 30% of their maximal strength for 3-min duration. We measured BP during the maneuver in the contralateral arm. The vasoconstrictor response was calculated by calculating the difference between baseline diastolic blood pressure (DBP) and DBP at the end of 2 min during the maneuver (Low, 2004).

### Statistical Analysis

Data were subjected to a normality test. Height, weight, BMI, HR, BP, and BRS values passed normality test and are expressed as mean ± standard deviation (SD) and compared using unpaired Student’s *t*-test. Short-term HRV parameters and autonomic reactivity test parameters did not pass the normality test and are expressed as median [interquartile range (IQR)] and compared using the Mann-Whitney U test. *p* <0.05 was considered to be statistically significant.

## Results

The anthropometric data including height (cm) (athletes – 159.26 ± 5.66, non-athletes – 160.55 ± 6.21), weight (kg) (athletes – 48.33 ± 5.09, non-athletes – 47.81 ± 5.06) and BMI (kg/m^2^) (athletes – 19.05 ± 1.83 and non-athletes – 18.51 ± 1.19) were comparable.

Groups were comparable based on HR (beats per minute) (athletes – 71.20 ± 2.44, non-athletes − 72.50 ± 3.45), systolic blood pressure (mm Hg) (athletes – 108.83 ± 5.12, non-athletes − 109.60 ± 4.75) and diastolic blood pressure (mm Hg) (athletes – 87.67 ± 5.74, non-athletes – 87.83 ± 5.68).

**Figure 1:**
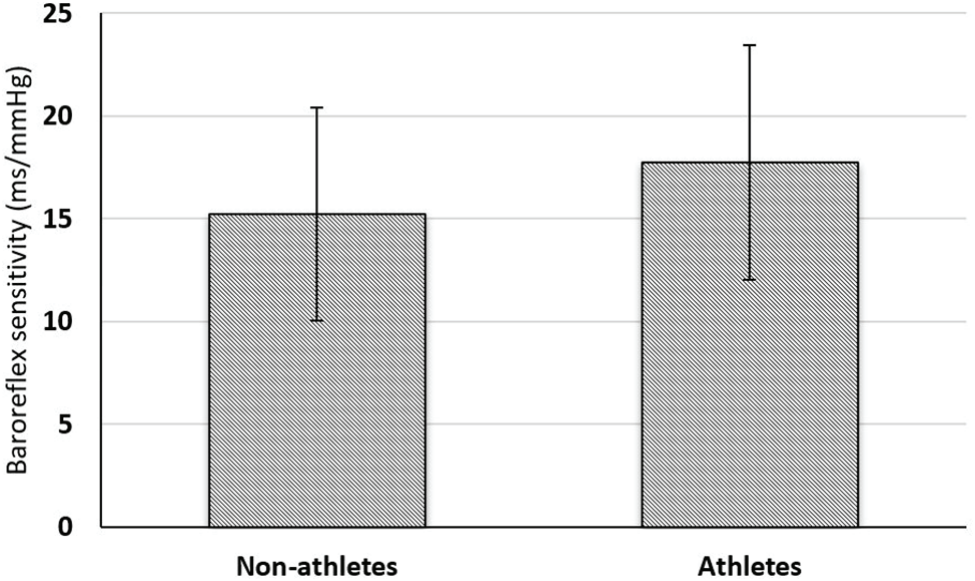
Comparison of baroreflex sensitivity between non-athlete (*n* = 30) and athlete boys (*n* = 30). Comparison of BRS (ms/mm Hg) between the groups was done using unpaired Student’s *t*-test. ^*^p < 0.05 is considered statistically significant.

Figure 1 shows that BRS is higher in athletes. However, the difference is not statistically significant.

Table 1 shows a comparison of time domain parameters between the groups. SDNN which denotes overall HRV is statistically higher in athletes. RMSSD (statistically significant), NN50, and pNN50, which denote high-frequency variations (parasympathetic activity), are higher in athletes.

**Table 1:** Short-term heart rate variability – time domain parameters.

Parameters	Non-athlete (*n* = 30)	Athlete (*n* = 30)	*p*
SDNN (ms)	67.20 (97.80)	88.40 (113.30)	0.006
RMSSD (ms)	80.85 (162.50)	108.05 (153.20)	0.034
NN50 (count)	122.50 (192.00)	142.50 (216.00)	0.119
pNN50 (%)	41.75 (55.90)	42.95 (70.40)	0.204

*Data presented as median (interquartile range). SDNN, standard deviation of all NN intervals; RMSSD, square root of mean of the sum of the squares of differences between adjacent NN intervals; NN50, number of pairs of adjacent NN intervals differing by more than 50 ms in entire recording; pNN50, number of adjacent NN intervals which differ by more than 50 ms. Comparison between the groups was done using Mann-Whitney U test. *p* < 0.05 is considered statistically significant*.

Table 2 shows a comparison of frequency domain parameters between the groups. Total power is significantly higher in athletes, and absolute values of powers in all frequency ranges are significantly higher in athletes; this denotes that short-term heart rate variability is higher in athletes. Based on the normalized units (HFnu and LFnu), relative parasympathetic tone is higher and relative sympathetic tone is lower.

**Table 2:** Short-term heart rate variability – frequency domain parameters.

Parameters	Non-athlete (*n* = 30)	Athlete (*n* = 30)	*p*
VLF (ms^2^)	128.50 (1219.00)	159.00 (3310.00)	0.301
LF (ms^2^)	1475.50 (7635.00)	1839.50 (3681.00)	0.037
HF (ms^2^)	1613.50 (8855.00)	3372.00 (8141.00)	0.012
Total power (ms^2^)	3354.50 (12561.00)	6130.00 (10167.00)	0.006
LF/HF ratio	0.866 (3.84)	0.631 (1.86)	0.030
LF (nu)	46.42 (59.52)	38.69 (54.85)	0.032
HF (nu)	53.57 (59.52)	61.30 (54.85)	0.032

*Data presented as median (interquartile range). VLF, very low frequency (0.003–0.04 Hz); Total power, the variance of NN intervals over the temporal segment; LF, power in low-frequency range (0.04–0.15 Hz); HF, power in high-frequency range (0.15–0.4 Hz); LF (nu), LF power in normalized units [LF/(TP − VLF) × 100]; HF (nu), HF power in normalized units [HF/(TP − VLF) × 100]; LF/HF ratio, ratio LF (ms^2^)/HF (ms^2^). Comparison between the groups was done using Mann-Whitney U test. *p* < 0.05 is considered statistically significant*.

Table 3 shows comparison of autonomic reactivity tests. Orthostatic stress 30:15 ratio is predominantly parasympathetic dependent, while E:I ratio is purely parasympathetic dependent and diastolic rise during isometric handgrip test denotes sympathetic reactivity. 30:15 ratio and E:I ratio are significantly higher in athletes, while diastolic rise during isometric handgrip test is comparable between the groups. Hence, parasympathetic reactivity is higher in athletes while sympathetic reactivity is comparable between the groups.

**Table 3:** Comparison of autonomic reactivity tests between the groups.

Parameters	Non-athlete (*n* = 30)	Athlete (*n* = 30)	*p*
Orthostatic stress (30:15 ratio)	1.42 (0.68)	1.51 (0.69)	0.025
Forced timed breathing (EI ratio)	1.30 (0.36)	1.40 (0.60)	0.043
Isometric handgrip test	17.00 (2.00)	18 (2.00)	0.293

*Data presented as median (interquartile range). 30:15 ratio, the ratio of the longest RR interval recorded after standing to the shortest RR interval recorded during supine position; EI ratio, the ratio of longest RR interval during expiration to shortest RR interval during inspiration; Isometric hand grip test, difference in baseline diastolic blood pressure and diastolic blood pressure after an isometric exercise. Comparison between the groups was done using the Mann-Whitney U test. *p* < 0.05 is considered statistically significant*.

## Discussion

In the present study, we have assessed BRS and autonomic function in adolescent athlete and non-athlete boys. Age, obesity, and gender are the major factors known to influence BRS (Monahan, 2007; Skrapari et al., 2007; Adoor et al., 2018) and autonomic function (Sharma et al., 2015). To minimize the effects of these factors, we have recruited only age-matched male participants. Further, both groups were comparable based on BMI. Students are from the same locality, socioeconomic background, and studying in CBSE schools. Hence, we believe that the difference between groups due to environmental influence on autonomic function would have been minimal.

Baroreflex sensitivity is measured in terms of change in the interbeat interval (IBI) in milliseconds per unit change in blood pressure in mm Hg. The increase or decrease in IBI in response to decrease or increase in blood pressure by baroreflex might be through any one limb (sympathetic or parasympathetic) of the autonomic nervous system or both together. However, the rapid change is feasible mainly through the parasympathetic limb. Baroreflex sensitivity reflects the complex interaction between the vascular and autonomic function to manage the blood pressure fluctuations of daily life within normal levels. In our study, we observed a trend toward higher BRS in the athlete group. This finding is supported by previous exercise intervention studies done in healthy adults (Iwasaki et al., 2003) and healthy seniors (Okazaki et al., 2005). Higher BRS might be due to the effect of exercise on enhancing the distending capacity of blood vessels and signal transduction in baroreceptors (Monahan et al., 2000; Tanaka et al., 2000a,b) or by improved integration at central cardiovascular centers (Iwasaki et al., 2003).

Reduced BRS is shown to be associated with high blood pressure (Chung and Bruehl, 2008) and resetting of baroreceptor working range to a higher level is seen in hypertension (Krieger, 1988). In our study, even though the BRS is lower in the non-athlete group, the systolic or diastolic blood pressure was comparable with that of the athlete group. We hypothesize that in our study, the level of decrease in BRS is not enough to bring a change in blood pressure or the relation between reduced BRS, high blood pressure, and physical activity observed in previous studies might be due to other unknown mechanisms. In support to our argument, Dutoit et al. have stated that there is no correlation between cardiac baroreflex sensitivity and sympathetic baroreflex sensitivity within healthy young humans particularly in males and BRS measured may not reflect the buffering capacity of the baroreceptor mechanism (Dutoit et al., 2010). Short-term blood pressure regulation is by a neural mechanism such as baroreceptor reflex, and long-term control is by renal mechanisms. The decrease in BRS might manifest only during a demand such as an exercise in early stages and requires some more years or an additional defect in renal mechanism to get reflected in resting blood pressure. It has been shown by Meredith et al. that the effect of physical activity is mainly on renal sympathetic and not on cardiac sympathetic activity (Meredith et al., 1991). However, many authors believe that the reduction in blood pressure is mainly due to a reduction in sympathetic vasomotor tone (Brown et al., 2002; Laterza et al., 2007; Fu et al., 2010).

The relation between cardiac sympathetic overactivity and its association with cardiovascular diseases such as hypertension and heart failure are well established. Sedentary individuals are shown to have higher sympathetic tone even at rest and higher reactivity to any stress (Monda et al., 2011). However, in our study, we observed only a relative increase in sympathetic activity (LFnu and LF/HF ratio) in the non-athlete group. Based on recent publications, the correlation between LF power and sympathetic nerve activity is not clear (Houle and Billman, 1999; Billman, 2013) and, hence, we would not be able to comment about the sympathetic activity in our study based on LFnu and LF/HF ratio alone. Further, LF power was significantly more in athletes than non-athletes. Studies have shown that selective parasympathectomy or cholinergic antagonists reduce LF power by 50% (Akselrod et al., 1981; Randall et al., 1991). Hence, we could hypothesize that the increase in LF power observed in athletes could be due to the parasympathetic component of ANS rather than by sympathetic component. We also did not observe any significant difference in diastolic blood pressure changes to isometric handgrip test between the groups. A decrease in sympathetic activity is expected in physically active individuals as repeated activation of the sympathetic system during each session of exercise is shown to attenuate its response (Grassi et al., 2000; Fraga et al., 2007; Fu et al., 2010). Physical activity is shown to reduced sympathetic activity by reducing weight in overweight or obese individuals (Joyner and Green, 2009) or by increasing muscle mass (Kraus and Levine, 2007) and thereby reducing insulin-mediated sympathetic activity (Julius et al., 1991; Baron et al., 1993). Since the BMI in our study is comparable between the groups, the effect of physical activity might have been minimal in the sympathetic limb of the ANS.

Further, Rosenwinkel et al. have stated that the effect of exercise training on heart rate is mainly due to parasympathetic activity and the sympathetic activity has only minimal impact (Rosenwinkel et al., 2001). This is supported by our findings from other cardiac autonomic function tests; we observed higher total power and SDNN, which denotes overall increase in heart rate variability; we also observed higher HF power and RMSSD in athletes, which are indicative of resting parasympathetic tone and higher 30:15 ratio and E:I ratio in athletes, which indicates higher parasympathetic reactivity. This goes hand in hand with available evidence that has shown that physical activity enhances parasympathetic activity. Hence, the improved BRS might be due to change in parasympathetic tone or parasympathetic reactivity predominantly. However, in contrast, Bronwyn et al. have argued that exercise training mainly acts on the sympathetic limb of the ANS to improve BRS and it even reduces the parasympathetic contribution on BRS (Kingwell et al., 1992). In our study, we were able to bring out the parasympathetic effect of physical activity, and we were not able to comment on the sympathetic component.

## Conclusion

Regular physical activity might be beneficial to improve the autonomic tone, which can further improve cardiovascular health as evidenced by higher vagal modulation among athletes than age-matched non-athletes.

## Limitation

Lack of significant difference in baroreflex sensitivity and other parameters might be due to small sample size. Further, the non-athlete group is not a sedentary population; they were also physically active as part of school co-curriculum. Athlete group consisted of students with various levels of training and competitiveness; this could have influenced BRS and HRV values. We have not quantified the level of physical activity intensity, and its influence on HRV was not studied. The parameters that could have influenced the autonomic activity such as diet, academic stress, and environmental influence were not measured.

## Future Perspectives

A direct measurement such as that of muscle sympathetic nerve activity would help us in determining the effect of physical activity on the sympathetic nervous system.

## Data Availability

The datasets generated for this study are available on request to the corresponding author.

## Author Contributions

SS, VS, VA, RR, and AG conceived and designed the analysis. SS, VS, RR, and AG collected the data and contributed data or analysis tools. SS, VS, and RR performed the analysis and wrote the paper.

### Conflict of Interest Statement

The authors declare that the research was conducted in the absence of any commercial or financial relationships that could be construed as a potential conflict of interest.
